# Stellate ganglion, inflammation, and arrhythmias: a new perspective on neuroimmune regulation

**DOI:** 10.3389/fcvm.2024.1453127

**Published:** 2024-09-12

**Authors:** Qiulian Lei, Zefei Jiang, Yu Shao, Xinghong Liu, Xiaoping Li

**Affiliations:** ^1^ School of Clinical Medicine, Chengdu University of Traditional Chinese Medicine, Chengdu, Sichuan, China; ^2^ Acupuncture and Tuina School, Chengdu University of Traditional Chinese Medicine, Chengdu, Sichuan, China; ^3^ Department of Cardiology, Sichuan Provincial People’s Hospital, University of Electronic Science and Technology of China, Chengdu, Sichuan, China

**Keywords:** stellate ganglion, inflammation, arrhythmias, neuroimmunity, clinical application

## Abstract

Current research on the stellate ganglion (SG) has shifted from merely understanding its role as a collection of neurons to recognizing its importance in immune regulation. As part of the autonomic nervous system (ANS), the SG plays a crucial role in regulating cardiovascular function, particularly cardiac sympathetic nerve activity. Abnormal SG function can lead to disordered cardiac electrical activity, which in turn affects heart rhythm stability. Studies have shown that excessive activity of the SG is closely related to the occurrence of arrhythmias, especially in the context of inflammation. Abnormal activity of the SG may trigger excessive excitation of the sympathetic nervous system (SNS) through neuroimmune mechanisms, thereby increasing the risk of arrhythmias. Simultaneously, the inflammatory response of the SG further aggravates this process, forming a vicious cycle. However, the causal relationship between SG, inflammation, and arrhythmias has not yet been fully clarified. Therefore, this article deeply explores the key role of the SG in arrhythmias and its complex relationship with inflammation, providing relevant clinical evidence. It indicates that interventions targeting SG function and inflammatory responses have potential in preventing and treating inflammation-related arrhythmias, offering a new perspective for cardiovascular disease treatment strategies.

## 1 Introduction

Arrhythmias have been a long-standing focus of cardiovascular disease research, with morbidity and mortality increasing with age and posing a significant burden on global public health (1, 2). Arrhythmias is not only an independent pathophysiological problem, but it can also trigger complex hemodynamic disorders, leading to insufficient heart pumping function and significantly increasing the risk of stroke and heart failure (HF). For example, in patients with atrial fibrillation (AF), irregular atrial contractions can cause blood to stagnate within the atria, forming clots that increase the likelihood of stroke (3–5). In addition, this hemodynamic disorder can seriously affect the patient's quality of life, manifesting as the aggravation of symptoms such as limited physical activity, dyspnea, and fatigue, further promoting the progression of HF (6). Additionally, ventricular arrhythmias (VAs), especially ventricular fibrillation (VF), may lead to sudden cardiac death (SCD), further underlining the fatal nature of the disease (7–9).

ANS dysfunction, particularly due to overactivation of cardiac sympathetic nerves, is a key trigger of arrhythmias. This dysfunction can lead to uncoordinated electrical activity of the heart, manifested as significant and heterogeneous changes in atrial and ventricular electrophysiology, thereby inducing various types of arrhythmias, and even leading to life-threatening arrhythmias (10). In particular, overactivated sympathetic nerves can significantly increase the excitability of cardiomyocytes, leading to an increased heart rate and instability of electrical activity (11, 12). These changes are particularly pronounced in patients with underlying cardiovascular disease, increasing the risk of serious arrhythmias in these patients. Moreover, arrhythmias can also directly affect the heart rhythm regulation center in the brainstem, leading to an imbalance in heart rhythm regulation and making arrhythmias more likely to occur (13, 14). In this process, the SG, as the main source of sympathetic input to the heart, plays a crucial role in the regulation of cardiac rhythm, and its overactivity can lead to sympathetic neurogenic excessive activity, thereby increasing the risk of cardiac arrhythmias (15, 16).

Recent association studies have shown that the incidence of SG involvement is increased in patients with arrhythmias, with a large number of inflammatory cell infiltrations, glial cell activation, and neuronal remodeling observed in the SG of these patients (17). This suggests a close relationship between SG and inflammation. However, it remains unclear whether SG inflammation is a direct cause of arrhythmias or merely a secondary or contributing factor. Despite the lack of clear evidence for a causal relationship, SG, inflammation, and arrhythmias may interact through neuroimmune mechanisms, jointly leading to sympathetic overactivity and thereby increasing the risk of arrhythmias. This article explores the relationship between SG, inflammation, and arrhythmias, clarifies the neuroimmune regulatory mechanisms underlying arrhythmias, and provides a feasible approach for the clinical management of this condition.

## 2 Pathologic manifestations of stellate ganglion inflammation and cardiac arrhythmias

One report found that patients with SCD had significantly higher percentages of left stellate ganglion (LSG) inflammation compared with patients without SCD (18), suggesting that LSG inflammation is common in SCD and may be a factor in sympathetic hyperactivity (19). In patients with long QT syndrome and catecholaminergic polymorphic ventricular tachycardia (VT), the SG also showed significant inflammatory infiltration and a significantly higher number of CD3+ and CD8+ *T* cells than in healthy controls. This suggests that T cell-mediated ganglion cytotoxicity may increase adrenergic activity and exacerbate electrical instability in these patients (20, 21). Furthermore, studies of patients with cardiac sympathetic denervation due to cardiomyopathy and refractory VAs have shown that the SG exhibits inflammation, oxidative stress, and satellite glial activation. These changes may lead to efferent sympathetic hyperactivity and dysfunction (17, 22). All of this clinical evidence suggests an association between SG inflammation and the development of arrhythmias. However, there is no conclusive evidence for a causal relationship between SG, inflammation, and arrhythmias; instead, they appear to be part of a complex interacting process.

In addition to clinical manifestations, this phenomenon has also been observed in animal models. Myocardial infarction (MI) is considered to be a progressive inflammatory process (23, 24). After infarction, significant transcriptomic changes occur in the SG and dorsal root ganglia, specifically involving the induction of genes related to inflammatory signaling and apoptosis, exhibiting features of inflammation and apoptosis (25). Notably, cell death signaling and p53-mediated pathways are particularly affected, with TUNEL staining showing increased apoptotic signaling in SG, along with concomitant neuronal cell loss after chronic infarction (26). It is well known that VAs are among the most common complications following MI (27, 28). This evidence suggests that the development of VAs after infarction may be related to inflammatory signaling and apoptosis in the SG.

Apart from the inflammatory response after MI, other pathological conditions of nervous system damage may also affect cardiac function. Diabetic cardiac autonomic neuropathy is a serious complication of diabetes, characterized by damage to the sympathetic and parasympathetic nerve fibers in the ANS, leading to hypertension, arrhythmias, silent MI, and SCD (29, 30). Animal studies have shown that inflammation caused by type 2 diabetes leads to inflammation-related expression profiles in the superior cervical ganglion and SG, including co-expression of mRNAs related to immune response and chemokines (31), as well as the asynchronous release of neurotransmitters of tyrosine hydroxylase (TH), exacerbating sympathetic nerve dysregulation (32).

Inflammation of the SG is not only related to the previously mentioned ANS dysfunction but may also aggravate the occurrence of arrhythmias through interaction with endogenous opioid peptides, such as Nociceptin/Orphanin FQ (Noc). Studies have shown that during MI, Noc expression is upregulated in the SG and increases the risk of arrhythmias by shortening the action potential duration (APD) of cardiomyocytes (33, 34). In a rat model of sepsis, the NOP signaling pathway, the receptor for Noc, was significantly upregulated in the SG, suggesting that the release and potency of Noc are increased in the septic state. This process not only activates downstream signaling pathways, leading to the release of inflammatory mediators and further exacerbating cardiovascular damage, but may also directly lead to the occurrence of arrhythmias by inducing or exacerbating abnormalities in myocardial electrical activity (35, 36). Recent findings further highlight the close relationship between the SG and inflammation, suggesting that these interactions may contribute to autonomic dysfunction, particularly in the context of cardiac arrhythmias (37). Activation of NOP receptors may lead to arrhythmias by affecting the electrical activity of the myocardium. Therefore, these findings underscore the importance of neuroimmunomodulation as a potential therapeutic target, especially in the prevention and treatment of inflammation-related arrhythmias.

## 3 Interaction between the stellate ganglion, inflammation, and arrhythmias

### 3.1 Inflammation affects the stellate ganglion, causing arrhythmias

**Figure 1:**
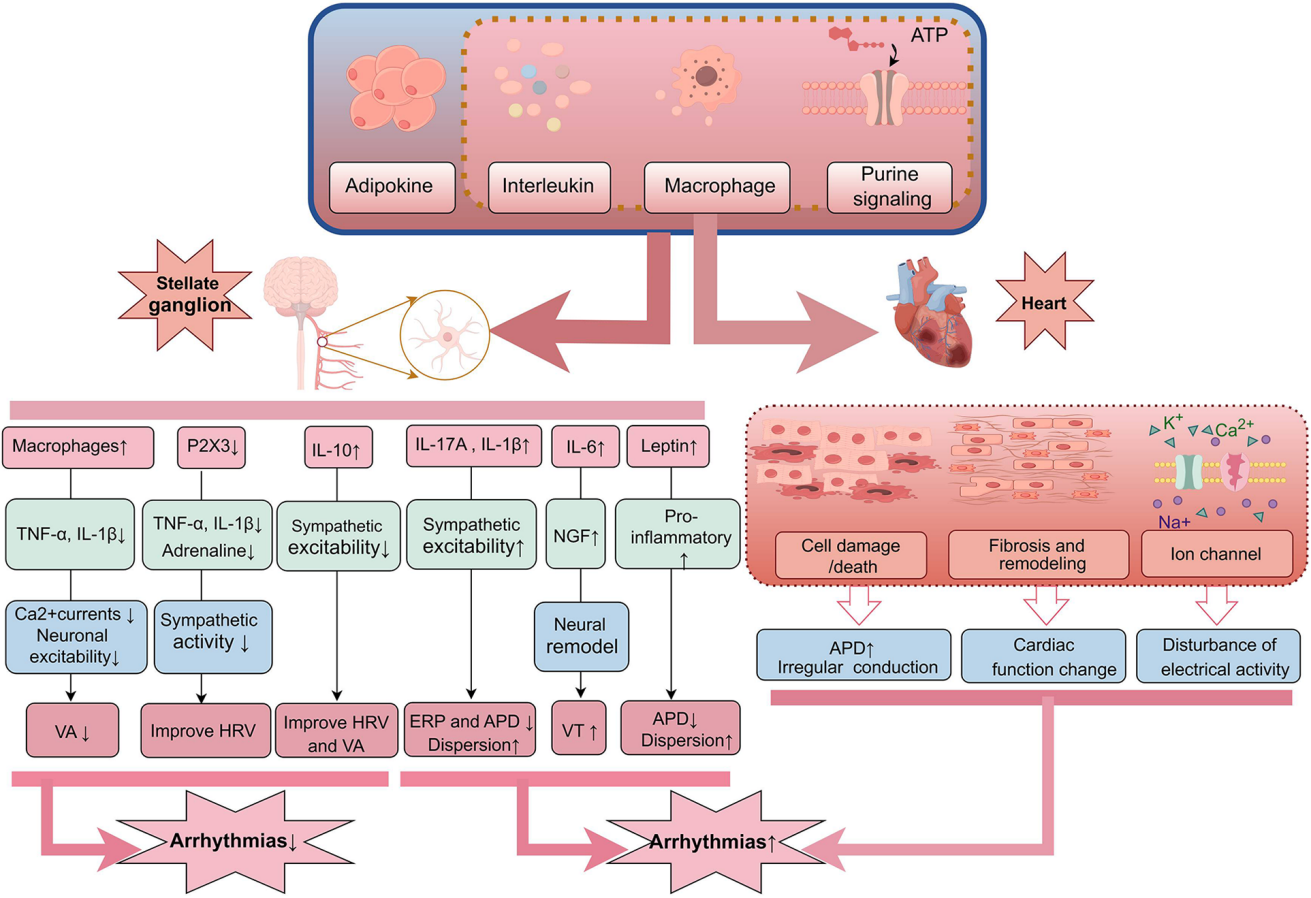
Inflammatory mediators released by the body during inflammation can affect both the nervous system and the heart. In the SG, an increase in certain pro-inflammatory factors such as IL-17A, IL-1β, IL-6, and TNF-α can enhance sympathetic nervous system excitability, leading to neuronal remodeling and shortening ERP and APD, which may increase the risk of VAs. Meanwhile, elevated IL-10 and reduced P2X3 may exert inhibitory effects on sympathetic excitation, aiding in the improvement of HRV and the occurrence of arrhythmias. Additionally, these inflammatory mediators can directly act on cardiac tissue, causing cellular damage, fibrosis, and influencing ion channel currents, thereby disrupting cardiac electrical activity and resulting in arrhythmias. SG, stellate ganglion; ERP, effective refractory period; APD, action potential duration; VAs, ventricular arrhythmias; HRV, heart rate variability.

Previous studies have shown that inflammation is related to the occurrence and severity of arrhythmias, and interleukins (IL), as classic inflammatory factors, are inextricably linked to the development of arrhythmias. Interleukins affect the electrical activity and structure of the heart through multiple mechanisms, including causing cardiomyocyte damage and fibrosis (38), affecting ANS function (39), or directly altering the electrophysiological properties of cardiomyocytes (40), thereby increasing the risk of arrhythmias. Studies have found that in the normal hearts of experimental dogs, injection of exogenous IL-17A can promote LSG remodeling, shorten the effective refractory period (ERP) and APD, upregulate the expression of neuropeptides and pro-inflammatory factors, and disrupt ventricular electrical stability via neuroimmunity (41) (Figure 1). It can also enhance LSG activity after acute myocardial ischemia (AMI) and induce post-ischemic VAs (42). Similarly, injection of exogenous IL-1β after acute ischemia in dogs can upregulate the expression of nerve growth factor (NGF) and cofilin. This upregulation significantly shortens ERP and APD, and promotes pathological neural remodeling in the LSG. The increase in NGF is considered an important factor in promoting the growth and survival of sympathetic neurons (43), while the upregulation of cofilin may lead to abnormal remodeling and dysfunction of neural structures (44). Together, these changes exacerbate the hyperexcitability of the ANS, thereby increasing the risk of VAs (45). IL-6 is an important neuroimmune inflammatory factor; sustained overexpression in the LSG can enhance neural activation, promote the expression of NGF, and activate the signal transducer and activator of transcription 3 (STAT3) and regulator of G protein signaling 4 (RGS4) pathways, mediating neural remodeling and increasing the risk of VAs after MI (46). In contrast, the anti-inflammatory factor IL-10, when overexpressed in the SG, can significantly inhibit sympathetic nerve activity, attenuate the myocardial inflammatory response, improve heart rate variability (HRV), and thereby inhibit the occurrence of VAs (47).

Lately, studies have gradually recognized that obesity is not only a risk factor for metabolic syndrome, but also plays an important role in the development of cardiovascular diseases, particularly arrhythmias. Obesity is a significant contributor to the pathophysiology of AF and its complications (48). Adipose tissue secretes a variety of pro-inflammatory, pro-fibrotic, and vasoconstrictive mediators, leading to hemodynamic changes, increased sympathetic nerve tone, and a low-grade chronic inflammatory state. These changes promote atrial remodeling, increase AF susceptibility, and elevate the risk of SCD (49, 50). Obesity can drive mast cell-mediated neuroimmune responses into a chronic inflammatory state, thereby activating inflammatory signaling pathways and increasing sympathetic nerve excitability, leading to sympathetic nerve damage (51, 52). Leptin, an adipokine, increases the incidence of ischemic VAs after binding to its receptor in the LSG. This effect may be related to the activation of leptin receptors in the LSG, where receptor activity is heightened due to the high expression of pro-inflammatory factors (53). Additionally, leptin activates mast cells, causing macrophages to polarize into a pro-inflammatory phenotype, thereby inducing VAs (54). Studies have found that the use of mast cell stabilizers can reduce the incidence of VAs after MI. This anti-allergic effect alters the communication between mast cells and cardiac autonomic nerves, suggesting that the IL-6 and *γ*-aminobutyric acid systems in the LSG may be involved in this process (55). The adipocyte-derived hormone lipocalin is closely related to the ANS and can regulate the sympathetic nervous system to exert a cardioprotective effect. Pretreatment of the LSG with lipocalin significantly inhibited its function and activity, stabilized electrophysiological properties, and suppressed ischemic VAs in rats. This effect was associated with the activation of downstream AMPK, PPAR*α*, and PPAR*γ* pathways, the inhibition of oxidative stress, and the reduction of pro-inflammatory cytokine levels after binding to related receptors (56). Overexpression of lipocalin reduced the expression of NGF and growth-associated protein 43 (GAP 43) in response to AMI, while reversing the upregulation of inflammatory factors and inhibiting sympathetic nerve remodeling to alleviate cardiac remodeling (56).

Macrophages play a vital role in the occurrence and development of arrhythmias. By interacting with cardiomyocytes and other immune cells to regulate the homeostasis of the cardiac environment, macrophages influence the occurrence and persistence of arrhythmias (57). Studies have shown that cardiac macrophages affect myocardial conductivity and self-regulation by secreting inflammatory factors and participating in the myocardial remodeling process (58). When the body responds to changes in the external environment, macrophages are activated and polarized, participating in the inflammatory process and exacerbating the occurrence of arrhythmias (59). Acute lung injury is a high-risk factor for arrhythmias. In rats with bleomycin-induced acute lung injury, an increase in macrophage-positive markers and macrophage activation was observed in the SG. This neuroinflammation increased the excitability of the SG and sympathetic nerve hyperactivity, leading to an increase in ventricular premature beats. However, anti-inflammatory treatment with minocycline attenuated this effect. In addition, enhanced excitability of SG neurons was found in co-cultures of macrophages pretreated with lipopolysaccharide (60, 61). HF is closely related to VAs-induced SCD, and the two together constitute the final stage of various heart diseases. The connection between VAs and HF is inseparable. In rats with chronic heart failure (CHF), nine inflammatory factors were detected in the SG, and TNF-α and IL-1β levels were increased (62). Depletion of macrophages in the SG using clodronate significantly reduced calcium currents and neuronal excitability, restored ventricular electrical activity heterogeneity, and reduced the incidence of VAs. Moreover, anti-inflammatory treatment with dexamethasone in the SG of rats with CHF reduced sympathetic hyperactivity, the incidence and duration of VAs, restored ventricular electrical activity heterogeneity, and reduced arrhythmias susceptibility (63). LSG ablation reduced the incidence of VAs induced by acute cerebral ischemia (64). This reduction was associated with a decrease in ventricular M1 macrophages and inflammatory cytokines after stellate ganglion ablation (SGB). Macrophage activation and polarization, as well as pro-inflammatory factors and sympathetic hyperactivity, increase arrhythmia susceptibility.

Purinergic receptors are essential in regulating inflammatory responses by modulating cell signaling, inflammatory mediator release, and immune cell activation (65). By participating in the inflammatory response and fibrotic processes, these receptors alter the normal pattern of cardiac electrical conduction, increasing the risk of arrhythmias (66). Specifically, P2X and P2Y receptors influence cardiac rhythm and conductivity by regulating electrophysiologic activity and calcium influx in cardiomyocytes (67, 68). Notably, abnormal expression of P2X4 receptors in the SG is closely related to inflammation. Parsnips can protect against high-fat-induced cardiac sympathetic neurogenic injury by downregulating P2X4 expression in the SG, inhibiting inflammatory factor expression in the serum, and decreasing cellular necroptosis-associated factors, thereby ameliorating obesity-induced sympathetic excitability (69). In the case of rat superior cervical ganglion P2X3 receptors and MI-induced sympathetic neuropathy, knocking down the P2X3 receptor in the SG reduced IL-1β and TNF-α expression and serum epinephrine concentration in diabetic rats. This attenuation of sympathetic nerve activity improved blood pressure and HRV (70). CpG oligodeoxynucleotides, which have cardioprotective and cytoprotective effects, can ameliorate sympathetic excitation and abnormal neuroglial signaling in diabetic rats (71). By decreasing upregulated NF-KB, P2Y12 receptor, TNF-α, and IL-1β in the SG, they reduce neuroinflammation and ferroptosis (72). Thus, modulating purinergic receptor activity presents a potential therapeutic strategy for the prevention and treatment of cardiac arrhythmias.

### 3.2 Stellate ganglion modulates inflammation and influences arrhythmias

After inflammation occurs, inflammatory genes in the SG are activated, leading to increased cell apoptosis and elevated neurotransmitter levels, which trigger neuroinflammation and subsequently affect the normal function and physiological processes of the ganglion. Studies have found that SGB can effectively inhibit the release of inflammatory mediators, control inflammation-related ANS, and reduce the degree of inflammation. In rats with thalamic hemorrhage, SGB inhibited the HIF-1α/NLRP3 signaling pathway, suppressed the excessive activation of microglia and astrocytes, reduced pro-inflammatory cytokines and oxidative stress, increased cerebral blood flow, and alleviated post-stroke pain and anxiety (73). Additionally, inhibition of central TNF-α converting enzyme in HF animals reduced TNF-α levels in the upper neck and SG, decreased neuroinflammation, and improved cardiac function (74). Clinical studies have shown that SGB can alleviate acute lung injury in patients with sepsis, reduce TNF-α and IL-6 levels, decrease NF-κB p65 expression, and increase the level of the anti-inflammatory factor IL-10 (75). LSG resection in rats with experimental autoimmune myocarditis was also found to activate the JAK2-STAT3 pathway, reduce inflammatory markers, and exert anti-arrhythmic effects (76). Therefore, by regulating the SG activity to attenuate inflammatory factors, SGB can modulate the occurrence and progression of inflammation, inhibit sympathetic nerve hyperexcitation, regulate post-injury dysfunction through neuroimmune regulation, and reduce the incidence of arrhythmias.

Previous studies have shown that AMI may induce the activation of microglia and sympathetic nerve cells, and that microglial activation may contribute to the activation of sympathetic nerve cells (77). Supporting this hypothesis, researchers found that noninvasive light-emitting diode therapy of the post-infarct SG significantly reduced premature ventricular contractions. This effect may be related to reduced microglial activation in the infarct border zone, which is associated with inflammatory and sympathetic-related factors. Therefore, this treatment suppresses inflammation and sympathetic activity, thus preventing MI-induced VAs (78). Targeting the LSG with low-intensity ultrasound results in attenuation of LSG function, reduced sympathetic activation, and significantly prolonged ERP, thereby reducing post-infarction VAs (79, 80). Optogenetic reduction of LSG function and activity significantly reduces myocardial infarct size, improves left ventricular (LV) function, and inhibits infarction-induced LSG sympathetic remodeling and LV fibrosis (81, 82).

With the advancement of science and technology, it has been discovered that certain new materials can directly affect the SG, potentially inhibiting sympathetic overactivation and alleviating arrhythmias. This provides new insights and potential therapeutic avenues for treating cardiac arrhythmias. For example, BTDBETA-COF-mediated hyperthermia can directly affect the function and neural activity of the SG, induce browning of white adipose tissue, improve the neuroinflammatory environment around the SG, and significantly downregulate inflammatory cell-related factors, as shown by transcriptome analysis. This helps inhibit ischemia-induced VAs (83, 84). Furthermore, carbon nanotubes (PC-CNT) significantly inhibited the neural activity and function of the LSG, alleviated arrhythmias, attenuated MI-induced VAs, and reduced NGF and c-fos levels, possibly by reducing neuroinflammation (85). Furthermore, local injection of endothelin-1 can activate LV systolic terminal flow, shorten the ERP, upregulate pro-inflammatory factors in the left ventricular systolic terminal flow, and aggravate the incidence of VAs caused by left descending coronary artery occlusion (86). This approach also reduces the density of nerve fibers in the infarct edge zone, weakens the remodeling of the SNS, and thereby improves the electrophysiological remodeling process.

## 4 Clinical applications of stellate ganglion

The SG are connected to key anatomical structures such as the heart, lungs, and gastrointestinal tract, and have a wide range of applications, including pain management, autonomic nerve modulation, and tumor therapy. Particularly, in the management of chronic pain conditions such as cancer or neuropathic pain, SGB or SG ablation techniques are often used to achieve long-term relief (87, 88). Additionally, the role of SG in the treatment of arrhythmias and inflammatory diseases has received increasing attention. For cardiovascular disease, SGB and permanent sympathectomy have been extensively studied. A large prospective multicenter study provided evidence for the efficacy and safety of SGB in the treatment of refractory electrical storm (ES) (89). Furthermore, a patient with cardiogenic shock was successfully treated with temporary mechanical circulatory support (MCS) and SGB, resulting in the restoration of normal cardiac rhythm and myocardial function (90). These techniques may positively impact cardiovascular health by modulating autonomic nerve activity and affecting heart rate (91). For example, percutaneous SGB has been used to treat refractory ES and sustained VT, particularly in patients on MCS (92, 93). In the 2017 AHA/ACC/HRS guidelines for the management of VAs, stellate ganglionectomy has been listed as a class IIb recommendation for patients with refractory VA (94).

Besides arrhythmia treatment, SGB has also demonstrated potential in regulating inflammatory responses. For example, studies have shown that SGB may have anti-inflammatory effects in diseases such as rheumatoid arthritis and inflammatory bowel disease (95). In patients with severe trauma, early use of SGB can reduce the release of proinflammatory cytokines, suggesting that it may play a role in modulating neuroimmune dysfunction after traumatic brain injury (96). In addition, SGB has been used to alleviate postoperative sleep disorders in patients undergoing surgery for gastrointestinal malignancies and stabilize perioperative hemodynamics (97).

Over the past few years, alongside traditional SGB, several innovative treatment methods are also being developed, including photodynamic therapy and low-intensity ultrasound therapy. These new technologies provide more options for the treatment of SG-related diseases, which is expected to improve efficacy and patients’ quality of life. However, these methods still need more clinical research and validation to confirm their safety, effectiveness and long-term results. Although these technologies may have anti-inflammatory effects in other clinical situations, the current evidence on whether the benefits of heart disease are achieved through anti-inflammatory pathways still needs further exploration. In summary, although SG and related technologies show broad prospects in the application of cardiovascular and inflammatory diseases, their specific benefits on heart disease through anti-inflammatory pathways still need further research and confirmation. At the same time, special attention should be paid to how to regulate cardiac electrophysiological activity through drugs or other methods to reduce the risk of arrhythmias. These studies also emphasize the importance of personalized treatment methods for specific conditions and the balance between effectiveness and safety during treatment (98–100).

## 5 Conclusion and outlook

The SG significantly affects the inflammatory process by regulating the release of neurotransmitters from the SNS, thereby acting on immune cells, inflammatory mediators, and related hormones. In an inflammatory state, the SG is not only affected by inflammation but may also lead to the activation of neurons and the release of additional neurotransmitters, thereby exacerbating the inflammatory response and progression. This increase in inflammation further exacerbates the risk of arrhythmias. Significantly, the cholinergic anti-inflammatory pathway plays a crucial role in regulating this process. Through the vagus nerve, the cholinergic anti-inflammatory pathway can inhibit the release of pro-inflammatory cytokines through the α7 subtype nicotinic acetylcholine receptor, effectively alleviating the increase in SG activity in inflammatory states (101, 102). This mechanism not only reduces the occurrence of inflammation-related arrhythmias but also highlights the importance of the neuroimmune system in cardiovascular disease. Although increased SG activity under inflammatory conditions may lead to abnormal excitation of the cardiac SNS, thereby triggering or exacerbating arrhythmias, current research shows that inflammation is not the only factor leading to SG activation. In addition, spontaneous arrhythmias caused by SG inflammation alone have not yet been observed, which means that more in-depth research on the relationship between the SG, inflammation, and arrhythmias is needed. A deeper understanding of these mechanisms can help us develop more effective clinical strategies, such as treatments targeting inflammation and interventions to modulate SG function, to prevent and control arrhythmias, thereby improving the quality of life and prognosis of patients with cardiovascular disease. These strategies can not only reduce the incidence of arrhythmias clinically, but also provide more precise treatment options for patients with complex cardiovascular diseases.
